# Cost-Effectiveness Analysis of Olaparib Maintenance Treatment for Germline BRCA-Mutated Metastatic Pancreatic *Cancer*

**DOI:** 10.3389/fphar.2021.632818

**Published:** 2021-04-20

**Authors:** Na Li, Huanrui Zheng, Yanlei Huang, Bin Zheng, Hongfu Cai, Maobai Liu

**Affiliations:** ^1^ Department of Pharmacy, Fujian Medical University Union Hospital, Fuzhou, China; ^2^ School of Pharmacy, Fujian Medical University, Fuzhou, China

**Keywords:** olaparib, cost-effectiveness, germline BRCA-mutated, metastatic, pancreatic cancer

## Abstract

**Background:** The PARP inhibitor olaparib has been shown to have clinical efficacy in patients with a germline BRCA mutation and ovarian or breast cancer. However, the high treatment cost associated with this drug limits its viability as a clinical treatment option. This work aims to evaluate the cost-effectiveness of olaparib as a maintenance treatment for metastatic pancreatic cancer from the perspective of the United States and China healthcare systems and provides valuable suggestions for clinical decision making.

**Method:** A three-state Markov model (progression-free, progressed disease, death) was constructed using TreeAge Pro 2020 software to evaluate the economic value of olaparib vs. placebo maintenance treatment for metastatic pancreatic cancer based on the clinical data derived from phase III randomized controlled trial (POLO, ClinicalTrials.gov number, NCT02184195). Total costs, quality-adjusted life years and incremental cost-effectiveness ratio were used as economic indicators for this analysis. A 5-years horizon and 5%/year discount rates were used. One-way sensitivity analysis and probabilistic sensitivity analysis (PSA) were performed to assess the model uncertainty.

**Results:** The incremental cost-effectiveness ratios (ICERs) of the use of olaparib vs. placebo in China and the United States were $6,694/QALY and $13327/QALY, respectively. All ICERs were far below the thresholds of $30829 in China and $50000 in the United States. Sensitivity analysis confirmed a stable economic advantage in the use of olaparib vs. placebo as maintenance therapy in China and the United States.

**Conclusion:** Olaparib was estimated to be more cost effective than placebo for the maintenance therapy of patients with a germline BRCA mutation and pancreatic cancer in China and the United States at thresholds of $30829 and $50000 per QALY, respectively.

## Background

Metastatic pancreatic cancer is particularly resistant to treatment and considered one of the worst prognostic malignancies (Rawla et al., 2019). Fewer than 10% of patients remain alive 5 years after the initial diagnosis (Von Hoff et al., 2013), and the improvement rate of survival from metastatic pancreatic cancer is relatively low compared with the steady increase in survival for most other cancers (Siegel et al., 2020). Because of the low diagnosis rate of pancreatic cancer, approximately 80% of all pancreatic cancer patients are already in advanced stages or distant metastases at the time of diagnosis (Conroy et al., 2011). Germline mutations in the tumor suppressors BRCA1, BRCA2, or both genes are linked to an increased risk of developing pancreatic cancers (Welcsh and King, 2001). Research shows that 4–7% of patients with pancreatic cancer have a germline BRCA mutation. The 3.2019 version of NCCN guidelines (National Comprehensive Cancer Network (NCCN), 2019) for pancreatic cancer recommends that all patients with pancreatic cancer should be sequenced to determine their BRCA1/2 mutation status.

The first-line treatment of pancreatic cancer has chosen to use traditional chemotherapy. FOLFIRINOX (consisting of oxaliplatin, irinotecan, fluorouracil, and leucovorin)/modified FOLFIRINOX“ and gemcitabine + albumin-bound paclitaxel” regimens are used in patients with advanced pancreatic cancer. And the second-line treatment can use the “5-FU + leucovorin + irinotecan liposome” regimen. The “gemcitabine + albumin-bound paclitaxel” regimen can also be used for second-line treatment when the patient is in good condition. Patients with pancreatic cancer who respond well to first-line chemotherapy, with the subsequent chemotherapy-free interval before disease progression usually ranging from 4 to 6 months. After disease recurrence, however, this chemotherapy-free interval becomes progressively shorter with the successive treatments given at each subsequent relapse. Maintenance treatment is a new concept in pancreatic cancer. Maintenance treatments that aim to extend progression-free and overall survival without compromising healthrelated quality of life are used in the management of many cancers and provide an opportunity to prolong responses.

The PARP inhibitor olaparib has been shown to have clinical efficacy in patients with a germline BRCA mutation and ovarian or breast cancer. PARP inhibitors can cause DNA damage to cancer cells by inhibiting the activity of PARP enzymes. DNA damage repair defects exist across tumor types, and the scope of application of PARP inhibitors is constantly expanding (Brown et al., 2017; Moore et al., 2018). A phase II clinical trial demonstrated responses to olaparib across different tumor types associated with germline BRCA1/2 mutations (Kaufman et al., 2015). A phase Ⅲ trial (Golan et al., 2019) indicated that, among patients with a germline BRCA mutation and metastatic pancreatic cancer, progression-free survival (PFS) is longer with maintenance using olaparib than with placebo (hazard ratio for disease progression or death, 0.53; 95% confidence interval [CI], 0.35–0.82; *p* = 0.004). Maintenance treatment is a new concept in pancreatic cancer. Maintenance treatments aim to extend PFS and overall survival (OS) without compromising health-related quality of life (HR-QoL). Olaparib maintenance therapy has been proven to be effective in patients with a germline BRCA mutation and metastatic pancreatic cancer. Because the adverse reactions of olaparib could be effectively controlled, its safety profile is good. However, although olaparib shows obvious advantages in the clinical efficacy of germline BRCA mutant metastatic pancreatic cancer, its high treatment cost limits its viability as a clinical treatment option.

This study evaluates the cost-effectiveness of olaparib vs. placebo as maintenance therapy for advanced recurrent BRCA mutant metastatic pancreatic cancer from the perspective of healthcare in China and the United States. The results provide a reference for the selection of safer, more-effective, and cost-efficient clinical treatment options for patients with BRCA mutation and metastatic pancreatic cancer in these two countries.

## Method

### Model Structure

**FIGURE 1:**
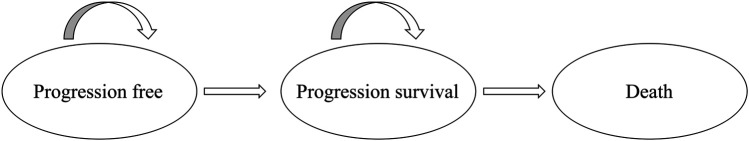
The Markov state transition model. Notes: The Markov model considers the transition states of metastatic pancreatic cancer. All patients start in the progression-free survival (PFS) state and receive treatment according to three treatment plans. Patients can enter the state of progression survival (PS) and subsequently move to the state of death.

A Markov model was developed to estimate the costs and treatment efficacy of metastatic pancreatic cancer and takes olaparib as a maintenance therapy compared with placebo within three mutually exclusive health states (Figure 1): “progression-free survival (PFS)” (initial state of patient until progression), “progression survival (PS)” (state after disease progression), and “death” (absorption state) (Gharaibeh et al., 2018). All patients entered the model from the PFS state; they could then either survive the PFS state or enter the PS state. Patients who transferred from PFS to PS could not recover their PFS state but continued to progress or die. As reported in the POLO trial (Golan et al., 2019), the patients had received at least 16 weeks of continuous first-line platinum-based chemotherapy for metastatic pancreatic cancer; the duration was unlimited as long as no evidence of disease progression was noted by the investigator during randomization. The maintenance trial intervention was initiated 4–8 weeks after the final dose of first-line chemotherapy was administered, and the patients were randomly assigned to receive maintenance olaparib tablets (300 mg twice daily) or the matching placebo at a 3 (92/154):2 (62/154) ratio.

The analysis was conducted from the perspective of the China and United States healthcare systems. Each model cycle represents 21 days, and the time horizon was 5 years. The primary outputs of this model were life-time health care costs, quality-adjusted life years (QALYs), and incremental cost-effectiveness ratios (ICERs). We adopted a 5% discount rate per year for costs and outcomes. Parametric survival curve fitting was performed in R (version 3.5.1) software, and the Markov model was developed and run in TreeAge Pro 2020.

### Effectiveness Parameters and Utility Estimates

The transfer probabilities of metastatic pancreatic cancer for the three health states were estimated based on the OS and PFS Kaplan–Meier (KM) curves of the POLO trial. The survival functions were used to calculate the transfer probabilities among the states. The GetData Graph Digitizer software package was used to extract probabilities from the curve published in the POLO trial (Table 1). A log-logistic distribution was fitted to the patient data because it provided the best fit compared with the Weibull, Exponential, Gompertz and Log-normal distributions. Fitting was also conducted according to the Akaike information criterion.

**TABLE 1:** Key model parameters.

	Shape	Scale	Distribution
PFS			
Olaparib	1.138	0.010	Log-logistic
Placebo	1.888	0.072	Log-logistic
OS			
Olaparib	1.403	0.014	Log-logistic
Placebo	1.757	0.006	Log-logistic

Abbreviations: PFS, progression-free survival; OS, overall survival.

QALYs were calculated by multiplying life-years by HR-QoL that is often referred to as utility (the health-state utility ranges from 0 [death] to 1 [complete health]). The health utility values of the three states were derived from previously published literature (Table 2). Regardless of the country assessed, the utility value was the same. Moreover, regardless of the therapy applied, the utility values of the PFS and PS states were the same (Attard et al., 2014; Zhou et al., 2016). A discount rate of 5% was applied to the QALY calculations.

**TABLE 2:** Health preference data.

	Utility	Distribution	Source
Olaparib (PFS)	0.85 [0.68–1.00]	Beta	Attard et al. (2014)
Olaparib (PS)	0.73 [0.584–0.876]	Beta	Attard et al. (2014)
Placebo (PFS)	0.85 [0.68–1.00]	Beta	Attard et al. (2014)
Placebo (PS)	0.73 [0.584–0.876]	Beta	Attard et al. (2014)

AbbreviationsPFS, progression-free survival; PS, progression survival.

### Cost Estimates

The costs involved in this study mainly included direct medical expenses, such as the cost of drugs, follow-up tests, management of adverse effects (AEs), best supportive care (BSC), and terminal care (Table 3). The unit price of olaparib in China was obtained from the Yaozhi network (XXX), and the cost of olaparib in the United States was obtained from the cost-effectiveness study of Guy et al. (2019). In the placebo group, the main costs were attributed to follow-up tests, BSC, and terminal care. The costs of follow-up tests, including biochemical tests, blood routine examination, and computerized tomography (CT), in China are obtained the actual charging standards of local medical institutions; in the United States, these costs are obtained from published studies (Guy et al., 2019; Liao et al., 2020). Follow-up tests costs were considered from the PFS state and calculated throughout the treatment process. All unit costs used in the base analysis are presented as United States dollars.

**TABLE 3:** Cost parameters.

		China				United StatesUS			
		Cost ($)	Range ($)	Dis	Source	Cost ($)	Range ($)	Dis	Source
Drugs costs	Olaparib/cycle	7,515.2	5,636.4–9,394	T	Yaozhi network (XXX)	13,886	10,414.5–17,357.5	T	Rawla et al. (2019)
AEs costs	Thrombocytopenia/unit	23.25	18.6–27.9	T	Zhou et al. (2016)	732.3	585.84–878.76	T	Guy et al. (2019)
	Neutropenia/unit	58.07	46.456–69.684	T	Zhou et al. (2016)	867.98	694.384–1,041.576	T	Guy et al. (2019)
	Fatigue/unit	0	0	T	Zhou et al. (2016)	0	0	T	Guy et al. (2019)
	Vomiting/unit	3.66	2.928–4.392	T	Zhou et al. (2016)	678.24	525.592–813.888	T	Guy et al. (2019)
	Anemia/unit	0	0	T	Zhou et al. (2016)	755.92	604.736–907.104	T	Guy et al. (2019)
Follow-up tests costs	Biochemical test/cycle	25.39	20.312–30.468	T	Hospital charges	72.43	50.70–94.16	T	Guy et al. (2019)
	Blood routine examination/cycle	3.53	2.824–4.236	T	Hospital charges	15.23	9.85–21.75	T	Guy et al. (2019)
	CT/cycle	94.58	75.664–113.496	T	Hospital charges	541.7	350.56–773.77	T	Guy et al. (2019)
BSC cost		117.12	93.69–140.54	T	Wu et al. (2014)	684.31	547.448–821.172	T	Gharaibeh et al. (2018)
Terminal care cost		1948.42	1,558.74–2,338.10	T	Wu et al. (2014)	85,904	55,592–122705	T	Guy et al. (2019)

Abbreviations: BSC, best support care; AEs, adverse events.

The unit costs of AEs in China and the United States were obtained from the cost-effectiveness studies of Zhou et al. (2016) and Guy et al. (2019), respectively. The cost of AEs was also considered in the placebo group. The POLO trial (Golan et al., 2019) revealed adverse events in the placebo group, but the incidence of these events was lower than that in the olaparib group. The adverse events included in our study included thrombocytopenia, neutropenia, fatigue, vomiting, and anemia. AEs-related costs were computed by multiplying the estimated incidence rate of each AE by the corresponding unit treatment cost. All unit AE costs used in the base analysis are listed in Table 3, and the incidence rates of each AE are listed in Table 4.

**TABLE 4:** Treatment-related adverse events.

AEs rates	Olaparib (%)	Placebo (%)
Thrombocytopenia	11	4
Neutropenia	23	12
Fatigue	60	35
Vomiting	20	15
Anemia	27	17

Abbreviations: AEs, adverse events.

BSC and terminal care are necessary in all strategies because of the lack of ideal replacement therapies and the high mortality of metastatic pancreatic cancer (Wu et al., 2014; Gharaibeh et al., 2018). After progression, the cost of BSC was the only cost included in the analyses. Terminal care costs were also included as a one-time cost in the final state. The above resource costs were obtained from previously published studies. The costs in the model are shown in United States dollars and based on the 2020 exchange rate (6.8985 yuan/United States dollar) (National Bureau of Statistics(NBS), 2020).

### Sensitivity Analysis

A series of sensitivity analyses were performed to explore how the results vary across a reasonable range. One-way sensitivity analyses were performed to assess the impact of individual parameters on the model. In the univariable sensitivity analysis, the parameters were assigned lower and upper limits obtained from credible intervals or a range of ±20% of the base-case value (Wan et al., 2019). Probabilistic sensitivity analyses (PSA) were conducted to explore uncertainties around key model inputs by varying them simultaneously. PSA was performed via Monte Carlo simulations with 1,000 iterations by using different distributions. The ranges and distributions of the parameters used in the sensitivity analyses are given in Tables 2, 3, respectively.

For the United States population in this study, $50000 was set as an acceptable threshold. Because of the lack of acceptable thresholds for the Chinese population, the World Trade Organization recommendations were adopted. Our study takes 3×China’s GDP per capita in 2019 as the threshold. According to the website of the National Bureau of Statistics, China’s GDP per capita in 2019 was 70,892 yuan ($10276.44). Thus, 3×GDP per capita is 212,676 yuan or $30892.

## Results

### Base Case Results

Compared with placebo, olaparib yielded increases in QALYs. In our analysis, the total costs of olaparib in the United States and China over a 5-year period were $208504 and $61477, respectively. The total costs of placebo use in the US and China were $91623 and $2,773, respectively, and the QALYs for the olaparib and placebo groups were 13.99 and 5.22, respectively. Patients treated with olaparib produced an additional 8.77 QALYs compared with patients on placebo maintenance therapy regardless of the country. The corresponding incremental costs over a 5-years horizon for olaparib vs. placebo in the United States and China were $116881 and $58704, respectively. Thus, the final ICERs in the United States and China were $13327 and $6,694, respectively (Table 5). These ICERs are far below the specified thresholds. Indeed, for China, the ICER was even less than 1×GDP per capita.

**TABLE 5:** Base case results.

Result	Olaparib	Placebo	ICER
China			
QALY	13.99	5.22	8.77
Total cost of regimen, $	61,477	2,773	58,704
ICER $/QALY	6,694		
The United States			
QALY	13.99	5.22	8.77
Total cost of regimen, $	208,504	91,623	116,881
ICER $/QALY	13,327		

Abbreviations: ICER, incremental cost-effectiveness ratio; QALY, quality-adjusted life year.

### Sensitivity Analyses

**FIGURE 2:**
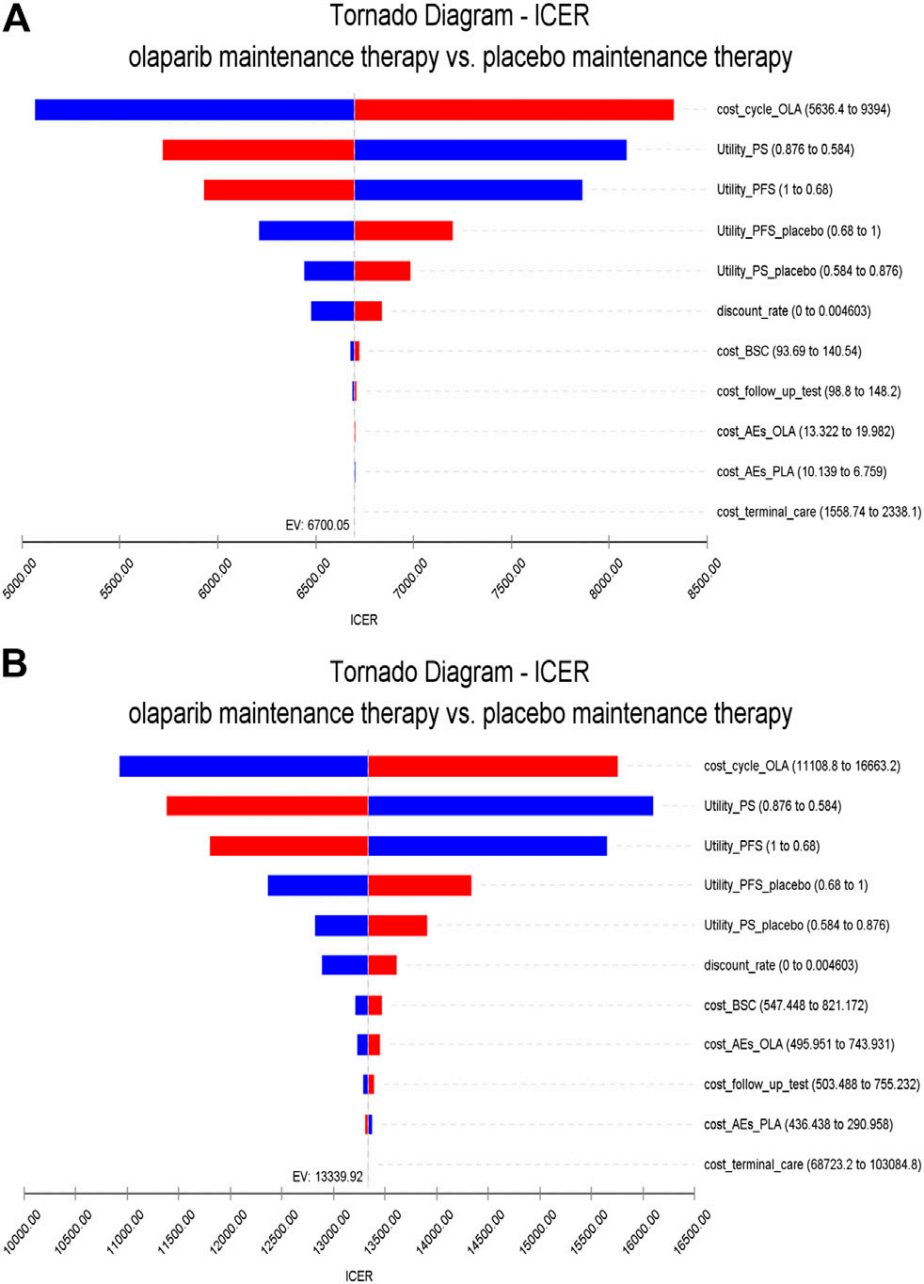
One-way sensitivity analysis. Notes: **(A)** is the result of China **(B)** is the result of the United States. The horizontal axis of the tornado graph indicates the range of influence of each factor on the result, and the vertical axis indicates the name of each uncertainty factor. The horizontal bar corresponds to the influence value of the factor on the result and the value of the factor itself. The factors are listed in descending order of their influence on ICER. Abbreviations: PS, progression survival; PFS, progression-free survival; BSC, best support care; AEs, adverse events.

The results of one-way sensitivity analyses are illustrated as tornado diagrams to describe the impact of the studied parameters on the model. The parameters were analyzed for single-factor sensitivity based on a reasonable range (±20%). In China, the cost of olaparib had the greatest impact on the ICERs obtained. The utility value of patients in the PFS state and discount rate were also factors affecting the outcomes of the ICERs. The ICERs in China was far below the threshold, as shown in Figure 2A. In the United States, among the factors analyzed, the cost of olaparib also had the greatest impact on ICERs. The utility value of patients in the PFS state and the cost of adverse events also revealed great influences on the ICER results, as shown in Figure 2B. Taken together, varying the key parameters in a sensible range had limited impact on the results.

**FIGURE 3:**
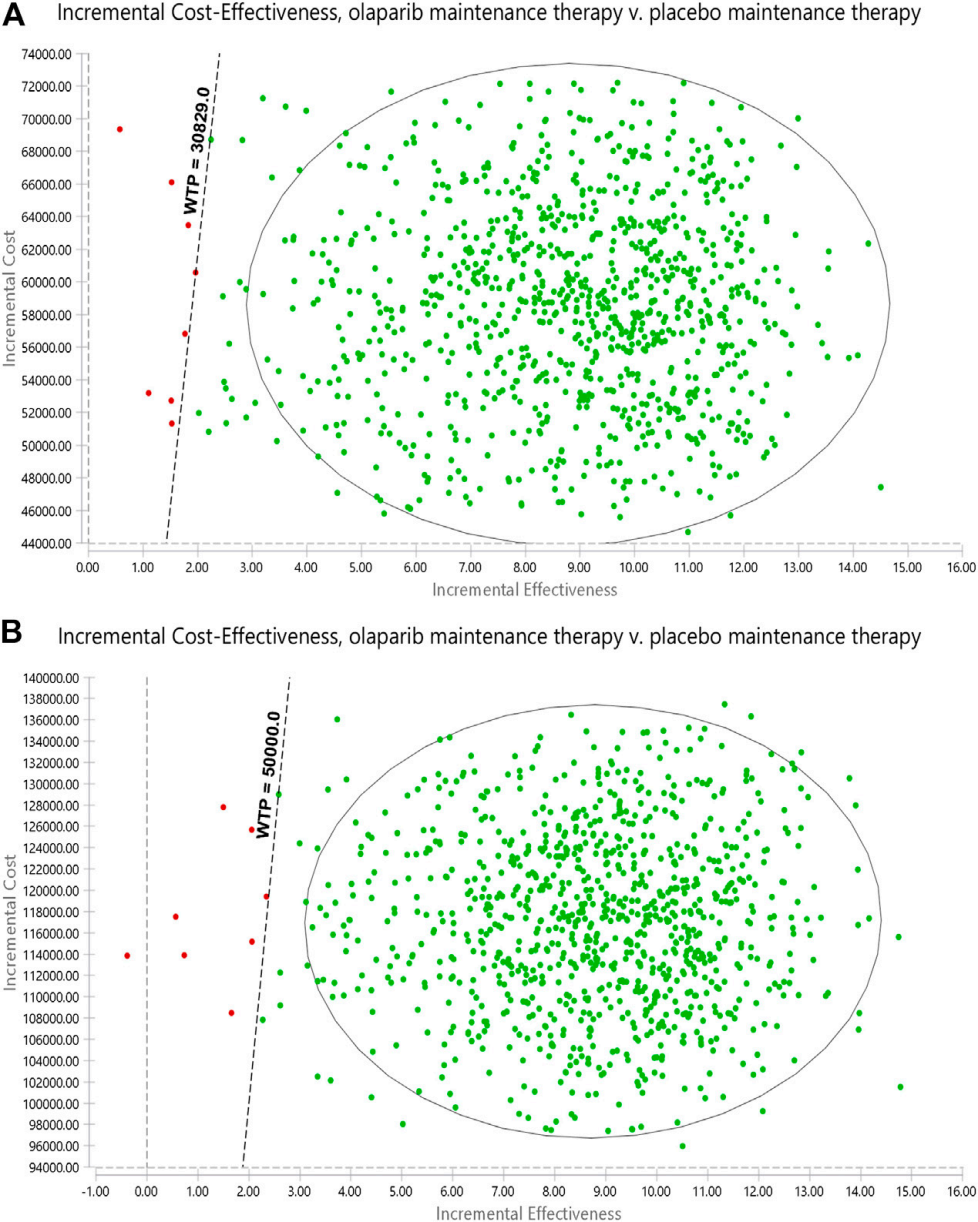
Probabilistic sensitivity analyses. Notes: **(A)** is the result of China, **(B)** is the result of the United States. The dot represents the results of the Monte Carlo simulation, and the ellipse represents the 95% confidence interval. The diagonal line represents the WTP. A dot falling below the diagonal line indicates that the test group has a cost effect compared with the corresponding control group. Abbreviations: WTP, willingness to pay; all costs are in United States dollars.

**FIGURE 4:**
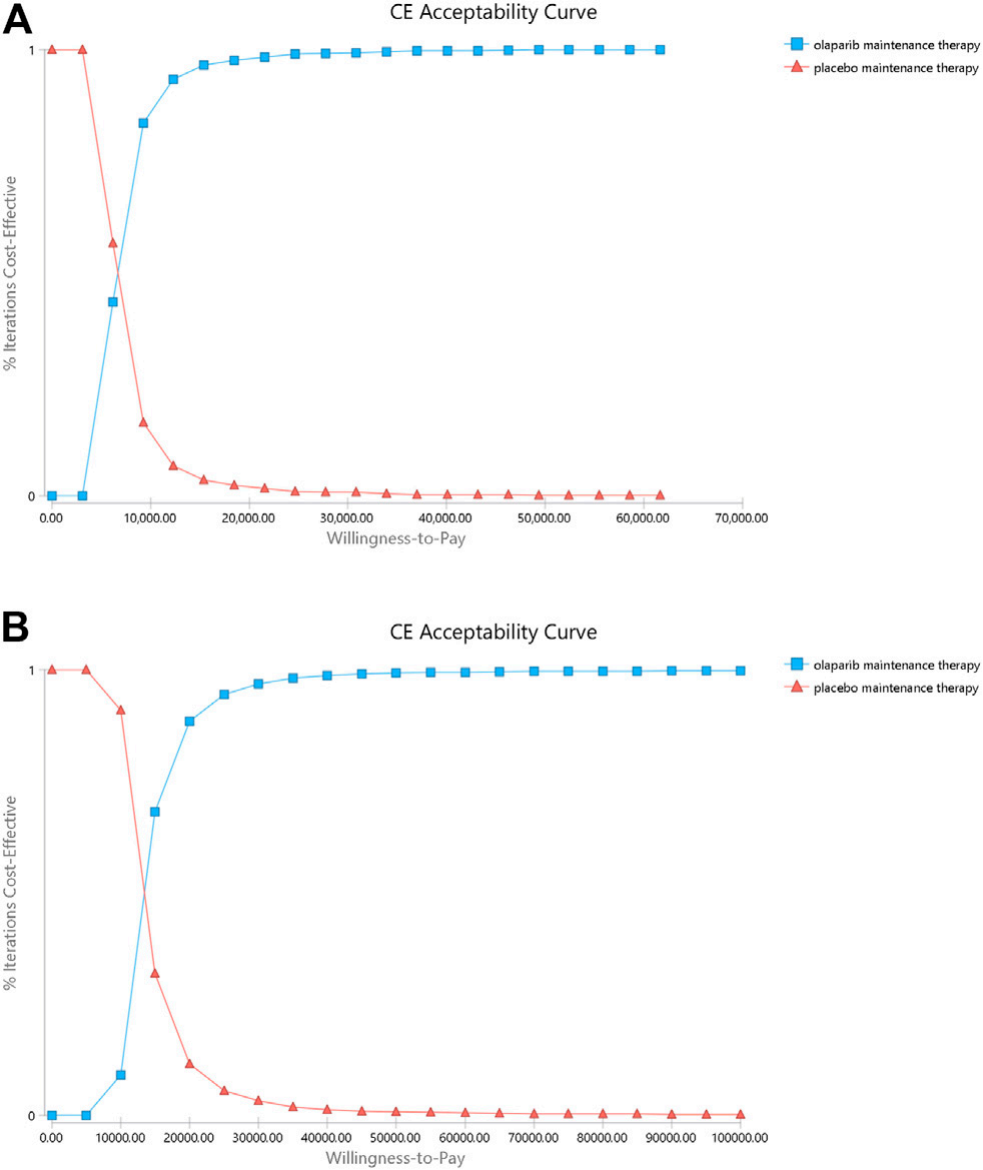
Cost-effectiveness acceptability curves (CEAC). Notes: **(A)** is the result of China **(B)** is the result of the United States. The CEAC is a curve used to indicate the probability of a drug being economical. The magnitude of the willingness-to- pay directly affects the cost effectiveness of the protocol. The acceptable curve shows the percentage of the cost-effectiveness of the simulation by using different treatment options. That is, the function of the relative change in cost effect is the ICER threshold change.

The range of values determined from the two countries and their respective distributions were simulated 1,000 times by using the Monte Carlo model. The PSA results are illustrated as a cost-effectiveness acceptability curve (Figure 3). In China, olaparib was cost-effective in 93.3% at a willingness-to-pay threshold of $12332 per QALY, olaparib was cost-effective in 98.3% at a willingness-to-pay threshold of $21580 per QALY, olaparib was cost-effective in 99.3% at a willingness-to-pay threshold of $30829 per QALY. In the US, olaparib was cost-effective in 88.4% at a willingness-to-pay threshold of $20000 per QALY, olaparib was cost-effective in 96.7% at a willingness-to-pay threshold of $30000 per QALY, olaparib was cost-effective in 98.7% at a willingness-to-pay threshold of $40000 per QALY, olaparib was cost-effective in 99.2% at a willingness-to-pay threshold of $50000 per QALY. The results of PSA are shown in Figure 4. In the United States and China, compared with placebo, olaparib revealed 99.5 and 99.3% scattering, respectively, on the willingness-to-pay curve.

## Discussion

Metastatic pancreatic cancer is a highly fatal disease characterized with limited therapeutic options and poor survival. The financial burden of this type of cancer is considerable. When first-line treatment fails, the disease enters the progressed stage, and very few effective treatment drugs may be used for treatment. Therefore, extending the PFS after first-line chemotherapy is of great importance. Primary analysis of the POLO trial (Golan et al., 2019) showed that patients who have a germline BRCA mutation and metastatic pancreatic cancer that had not progressed during first-line platinum-based chemotherapy have significantly longer PFS with olaparib than with placebo. The present study is the first to the analyze the cost-effectiveness of olaparib and placebo as maintenance regimens in metastatic pancreatic cancer. This study was carried out using the latest data from existing clinical studies. At present, only olaparib is currently used for maintenance treatment for metastatic pancreatic cancer. Maintenance treatment of patients with olaparib can significantly prolong their PFS and shows significant benefits compared with patients who do not use olaparib.

Our model demonstrated that, compared with placebo, olaparib produces an increment of 8.77 QALYs at incremental costs of $116881 and $58704 in the United States and China, respectively. ICERs of $13327 and $6,694 were obtained in the US and China, respectively. One-way sensitivity analyses using ±20% as a range boundary revealed that the main driver of ICER is the cost of olaparib regardless of the country. Because olaparib is very expensive, a slight change in the cost of the drug could cause significant effects on the ICERs obtained. However, the relationship between the ICERs and thresholds remained unchanged no matter lowered or upped values of key parameters. The utility of the PS state also has a significant influence on the ICERs because this state occupies a larger proportion of the patients’ OS time compared with the two other states. PSA indicated that olaparib may be more cost effective than placebo. These findings reveal that olaparib maintenance therapy is suitable for use in clinics when price and efficacy are taken into account simultaneously.

While the United States FDA officially approved olaparib as a maintenance treatment for patients with pancreatic cancer and inherited BRCA mutations after first-line platinum-containing chemotherapy on December 30, 2019, the drug has not been approved in China. The POLO clinical trial is the first and only phase III clinical study describing successful precision treatment based on biomarkers for metastatic pancreatic cancer. Olaparib was developed for the targeted treatment of pancreatic cancer. Research on the economics of the use of olaparib in the treatment of germline BRCA mutant metastatic pancreatic cancer is lacking. Our analysis is based on the healthcare system perspectives of China and the United States and takes into account the costs associated with drug treatment, disease detection, and adverse reaction management, as well as the different economic capacities of patients. Analyzing the Chinese population and the American population to understand the different payment costs and willingness to pay of them, and the collected data are fairly comprehensive and comparative. This work represents a breakthrough in the economic evaluation of pancreatic cancer-targeting drugs and provides a reference for future analyses on the cost effectiveness of target drugs for the treatment of this type of cancer. It also provides a reference for effective and economical clinical treatment therapies for patients in China and the United States, which is more practical and innovative.

Our study has the following limitations. First, the data of this study were obtained from a clinical trial of metastatic pancreatic cancer patients who met the inclusion criteria in 119 sites in 12 countries rather than clinical trials specifically targeting the Chinese and United States populations. While the trial was large and well designed, our model is essentially reliant on the validity and universality of the trial, and any deviations in the trial may be reflected in our research results. Second, the control therapy used in our study was placebo therapy. The use of standard treatments with the same indications for comparison is generally recommended. However, according to the guideline (National Comprehensive Cancer Network (NCCN), 2020), only olaparib is currently recommended as a standard maintenance treatment for patients with metastatic pancreatic cancer. Thus, our use of placebo treatment as a control therapy is reasonable. Third, the utility value reflects the HR-QoL, which is a subjective experience and may vary greatly among individuals; providing an accurate value for this factor is difficult. We did not consider reductions in utility value in the event adverse reactions occur. While this estimation is not ideal, we performed sensitivity analyses, which demonstrated that the variation of utility values does not qualitatively change the results. Fourth, indirect costs, i.e., income loss caused by suspension of school and early death, among others, are difficult to estimate; thus, we did not include these factors in the model. Other cost components may need to be considered. However, in the sensitivity analyses, the factor with the greatest effect was the cost of olaparib. To avoid affecting outcomes, we expanded the range of treatment costs via one-way sensitivity analysis. The variation ranges of the drug cost used in the sensitivity analyses almost considered variations in the payers’ reimbursement ratio. Finally, the model used in this study was based on the simplified development of pancreatic cancer. Therefore, accurately describing the disease progression of individual patients may be difficult. However, in our study, using the Weibull and Log-logistic distributions to simulate and correct disease progression trends. Thus, the actual cost of disease and QoL of patients were reasonably estimated. Regardless of these limitations, however, the variables in the model did not affect the final results. Sensitivity analysis showed that probability, utility, and costs are unlikely to affect the final outcome.

## Conclusion

Olaparib maintenance therapy was estimated to be highly cost effective for the patients with a germline BRCA mutation and metastatic pancreatic cancer from the perspective of the United States and China healthcare systems at thresholds of $50000 to $30892 per QALY, respectively.

## Data Availability

The raw data supporting the conclusions of this article will be made available by the authors, without undue reservation.

## Abbreviations

QALYs, quality-adjusted life years; ICER, incremental cost-effectiveness ratio; PSA, probabilistic sensitivity analysis; PFS, progression-free survival; OS, overall survival. HR-QoL, health-related quality of life; PS, progression survival; KM, Kaplan-Meier; AIC, akaike information criterion; AEs, adverse events; BSC, best supportive care; CT, computerized tomography.
